# Combinatorial effects of ion channel mis-splicing as a cause of myopathy in myotonic dystrophy

**DOI:** 10.1172/JCI176089

**Published:** 2024-01-02

**Authors:** Larissa Nitschke, Thomas A. Cooper

**Affiliations:** 1 Department of Pathology and Immunology,; 2 Department of Integrative Physiology and Biophysics, and; 3 Department of Molecular and Cellular Biology, Baylor College of Medicine, Houston, Texas, USA.

## Abstract

Myotonic dystrophy type 1 (DM1) is an autosomal dominant disorder caused by an unstable expanded CTG repeat located in the 3′-UTR of the DM1 protein kinase (*DMPK*) gene. The pathogenic mechanism results in misregulated alternative splicing of hundreds of genes, creating the dilemma of establishing which genes contribute to the mechanism of DM1 skeletal muscle pathology. In this issue of the *JCI*, Cisco and colleagues systematically tested the combinatorial effects of DM1-relevant mis-splicing patterns in vivo and identified the synergistic effects of mis-spliced calcium and chloride channels as a major contributor to DM1 skeletal muscle impairment. The authors further demonstrated the therapeutic potential for calcium channel modulation to block the synergistic effects and rescue myopathy.

## The role of mRNA mis-splicing in myotonic dystrophy

Myotonic dystrophy (DM) is an autosomal dominant disease that affects the skeletal muscle, heart, gastrointestinal tract, and brain. It is the most common cause of adult-onset muscular dystrophy, clinically affecting 1 in 8,500 individuals. The effects on skeletal muscle include myotonia and progressive muscle weakness and wasting and are the primary cause of mortality (1). The more common form in the United States, DM type 1 (DM1), has a mutation frequency of 1 in 2,100 and is caused by an unstable expanded CTG repeat in the 3′-UTR of the DM1 protein kinase (*DMPK*) gene, with 50 to more than 4,000 repeats in affected individuals and 5 to 38 repeats in those unaffected (2, 3). The predominant disease mechanism reflects a gain-of-function process in which the expanded *DMPK* allele yields mRNA with CUG repeats. The CUG repeats bind and sequester the Muscleblind-like (MBNL) family of RNA binding proteins. The relative depletion results in a loss of MBNL activity, disrupting its function as a regulator of alternative splicing of hundreds of genes, many of which drive transitions from fetal to adult protein isoforms during postnatal development. The resulting loss of MBNL function leads to failure of the fetal-to-adult transitions. The expression of fetal protein isoforms that are unable to perform the functions required in adult tissues is a major cause of pathogenesis in the affected tissues (4–6).

**Figure 1:**
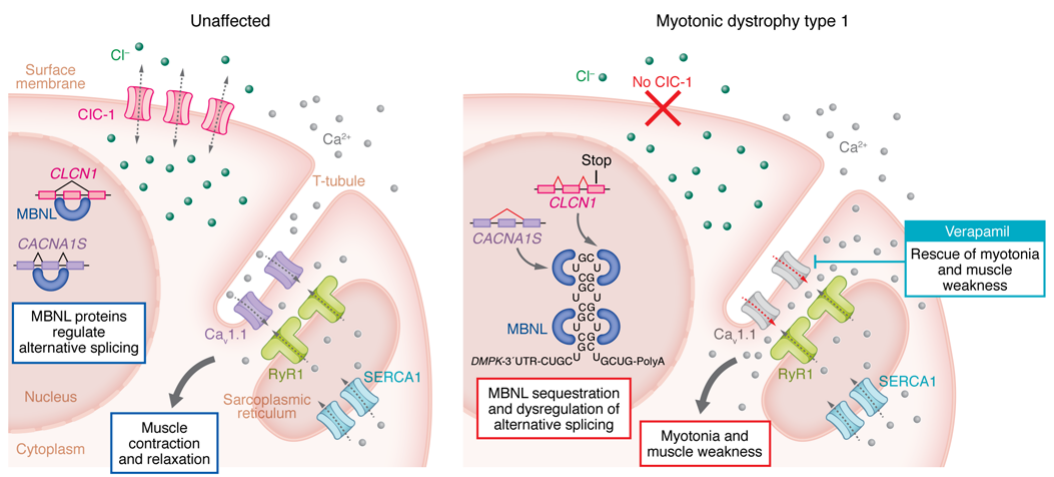
The combined effects of ion channels from mis-spliced RNA cause myotonia and muscle weakness in DM1. In unaffected muscle tissues, MBNL correctly regulates splicing of the alternative exons in *CLCN1* and *CACNA1S*. This process results in expression of the adult isoforms of the chloride and calcium channels, which, together with SERCA1 and RyR1, facilitate proper muscle contraction and relaxation. In DM1, MBNL sequestration on expanded CUG repeats causes the dysregulation of *CLCN1* and *CACNA1S* alternative splicing. The inclusion of a *CLCN1* fetal exon leads to a frameshift and premature stop codon, causing ClC-1 loss. Mis-splicing also leads to expression of the Ca_v_1.1 fetal isoform, which has an increased calcium current compared with the adult isoform. The combined loss of ClC-1 and expression of the fetal Ca_v_1.1 isoform leads to severe myopathy that can be rescued pharmacologically by blocking the calcium channel using verapamil.

A well-established example of mis-splicing in DM causes loss of the muscle-specific chloride channel ClC-1, leading to myotonia, a form of hypercontraction that affects muscle (7–9). ClC-1 protein expression is normally repressed in fetal skeletal muscle due to the inclusion of an out-of-frame fetal exon (e7a) in the mRNA, which leads to a premature stop codon. In adult muscle, the channel is expressed as a result of MBNL-mediated skipping of e7a during pre-mRNA processing. The flow of chloride ions in adult muscle maintains the strong electrochemical gradient primed for rapid depolarization for muscle contraction upon signaling from a motor neuron. Loss of MBNL activity in DM1 muscle results in e7a inclusion, loss of the ClC-1 channel, and chloride ion flow and a strong predisposition for depolarization independent of neuronal input, resulting in myotonia (Figure 1).

Transcriptome splicing analysis of DM1 skeletal muscle has identified hundreds of misregulated splicing events, including in most ion channels that regulate the normal flow of calcium ions involved in excitation-contraction coupling (10–12). Analysis of a cohort of patients with DM1 demonstrated that splicing misregulation of multiple alternative exons directly correlated with muscle weakness (11, 13). However, the specific molecular mechanisms for muscle wasting and weakness have remained unknown. The challenge after identifying the myriad of transcriptome changes that correlate with pathology is to define specific cause-effect relationships of individual genes. This is particularly challenging, given the possibility that many genes could contribute to a combinatorial effect that would be difficult to unravel if individual genes contribute incremental effects.

## Combined effects of mis-spliced Ca_v_1.1 and ClC-1 channels

In this issue of the *JCI*, Cisco and co-authors (14) used in vivo and ex vivo approaches to systematically test the combinatorial effects of four mis-spliced ion channels in a detailed analysis of skeletal muscle function. The postnatal splicing transitions of all four genes are conserved in humans and mice, all four are mis-spliced in DM1 skeletal muscle, and the DM1 splicing patterns of all four are reproduced by expression of CUG RNA repeats in skeletal muscle of an established DM1 mouse model (6, 11, 15). The authors used a frameshift mutation (16) to reproduce the loss of the ClC-1 channel function in mice and forced the DM1 splicing patterns of three calcium channels that are critical for excitation-contraction coupling: Ca_v_1.1, RyR1, and the sarcoplasmic reticulum Ca^2+^-ATPase SERCA1. To force expression of the DM1 protein isoforms of the three calcium channels, Cisco and colleagues used CRISPR/Cas9-mediated deletion of each of the alternative exons that are normally included in the mRNA to produce the adult isoform. The resulting mouse lines were bred to generate seven combinations of double-homozygous mice and one combination of the three calcium-handling genes. Notably, mice with the combination of Ca_v_1.1^Δe29/+^ ClC-1^–/–^ showed substantially reduced survival and severe muscle weakness consistent with DM1.

Previous work established that the fetal isoform of Ca_V_1.1, which has an enhanced calcium current compared with the adult isoform, aggravated muscle pathology in a DM1 mouse model expressing CUG RNA repeats (11). What was unknown was the dramatic synergistic effect on muscle pathology of increased calcium flow combined with the loss of chloride conductance. Using detailed analysis of multiple muscle function parameters ex vivo, Cisco and co-authors showed that increased calcium conductance potentiated the transient muscle weakness caused by loss of chloride conductance (14). Transient weakness resulted from the loss of ClC-1 channel function due to the runs of action potentials that caused myotonia and created a state of depolarization and the loss of excitability. The authors further showed that, in addition to muscle weakness, the increased calcium conductance enhanced myotonia, which was markedly worse in Ca_v_1.1^Δe29/+^ ClC-1^–/–^ mice than in ClC-1^–/–^ mice. Importantly, pharmacologic blockade of the Ca_v_1.1 channel by exposing the isolated muscle in the ex vivo bath to verapamil essentially eliminated both myotonia and transient weakness. These results demonstrated the synergistic effect of Cl^–^ and Ca^2+^ channels and the positive impact of blocking one of the contributing components (14) (Figure 1).

Given the positive effects of verapamil on transient weakness and myotonia in ex vivo experiments, the investigators tested long-term administration of verapamil to Ca_v_1.1^Δe29/+^ ClC-1^–/–^ mice. Remarkably, mice given 200 mg/kg/day exhibited rescue of weight loss, survival, and muscle function to near wild-type levels. Cisco et al. demonstrates how parsing out the specific components that produce severe pathology can reveal a relatively straightforward approach to block the synergistic effects toward a rescue (14).

## Conclusions and clinical implications

While the results support the modulation of calcium handling as a potential therapeutic target for skeletal muscle in DM1, as noted by Cisco and co-authors, the reality is more complicated (14). One of the challenges of treating a multisystemic disease is the possibility that a therapeutic for one cell type could have detrimental effects on another. DM1 features include cardiac condition abnormalities experienced by more than 50% of those affected, with at least 20% mortality due to fatal arrhythmias (17, 18). Verapamil is an effective medication for cardiovascular disease through its modulation of calcium flow in different cell types, but it is unclear whether the altered cellular physiology caused by DM1 will produce negative effects of verapamil in these cells. A next step will be to identify approaches to selectively modify calcium flow in skeletal muscle.

Interestingly, although the Ca_v_1.1^Δe29/+^ ClC-1^–/–^ bi-channelopathy mice were severely affected, they did not show all of the hallmarks of DM1 skeletal muscle, suggesting that the effect of CUGexp RNA disrupted the expression of additional genes that contribute to DM1 myopathy (14). Given that the broad range of myopathy hallmarks are induced in DM1 mouse models that express CUGexp RNA (19, 20), it will be of interest to determine the effect of modulating calcium channel activity on the myopathy induced by CUGexp RNA.

Current models propose that the progression of DM1 myopathy is due to the somatic instability of the CTG repeats that are particularly prone to expansion in skeletal muscle with aging. Cisco and colleagues showed that the Ca_v_1.1^Δe29/+^ ClC-1^–/–^ mice developed progressive myopathy, while the genetic changes causing the channelopathies were likely to be stable (14). This observation raises the interesting possibility that the channelopathies have a previously unrecognized contribution to the progressive nature of the myopathy.

The molecular understanding of DM1 pathogenesis established in the field has identified CUGexp RNA as a primary therapeutic target, leading to multiple promising approaches to degrade or otherwise neutralize the toxic RNA (21, 22). However, the mechanistic details that link the CUG RNA repeats to the causes of pathology in the multiple tissues affected remain to be determined. The results from Cisco et al. (14) are a major step toward teasing out the mechanisms that cause skeletal muscle pathogenesis and show how a deeper understanding of pathogenic mechanisms can lead to the identification of additional therapeutic approaches.

## Version 1 01/02/2024

Electronic publication
